# Automated classification platform for the identification of otitis media using optical coherence tomography

**DOI:** 10.1038/s41746-019-0094-0

**Published:** 2019-03-28

**Authors:** Guillermo L. Monroy, Jungeun Won, Roshan Dsouza, Paritosh Pande, Malcolm C. Hill, Ryan G. Porter, Michael A. Novak, Darold R. Spillman, Stephen A. Boppart

**Affiliations:** 1 Department of Bioengineering, University of Illinois at Urbana-Champaign, Urbana, IL USA; 2 Beckman Institute for Advanced Science and Technology, Urbana, IL USA; 3 Carle Foundation Hospital, Otolaryngology, Urbana, IL USA; 4 Carle Illinois College of Medicine, Urbana, IL USA; 5 Department of Electrical and Computer Engineering, University of Illinois at Urbana-Champaign, Urbana, IL USA

**Keywords:** Translational research, Paediatric research, Imaging and sensing, Biomedical engineering, Machine learning

## Abstract

The diagnosis and treatment of otitis media (OM), a common childhood infection, is a significant burden on the healthcare system. Diagnosis relies on observer experience via otoscopy, although for non-specialists or inexperienced users, accurate diagnosis can be difficult. In past studies, optical coherence tomography (OCT) has been used to quantitatively characterize disease states of OM, although with the involvement of experts to interpret and correlate image-based indicators of infection with clinical information. In this paper, a flexible and comprehensive framework is presented that automatically extracts features from OCT images, classifies data, and presents clinically relevant results in a user-friendly platform suitable for point-of-care and primary care settings. This framework was used to test the discrimination between OCT images of normal controls, ears with biofilms, and ears with biofilms and middle ear fluid (effusion). Predicted future performance of this classification platform returned promising results (90%+ accuracy) in various initial tests. With integration into patient healthcare workflow, users of all levels of medical experience may be able to collect OCT data and accurately identify the presence of middle ear fluid and/or biofilms.

## Introduction

Otitis media (OM) is a common infection of the middle ear in children, with combined direct and indirect annual costs estimated to be (US$) 4 billion.^1^ OM is a broad term for inflammation of the ear, which is further subdivided into specific disease states,^2^ including OM with effusion (OME),^1^ an accumulation of fluid within the middle ear cavity (MEC), and acute OM (AOM),^3^ an active infection, which may or may not include purulent and infected middle ear fluid. In either case, fluid accumulation may lead to hearing loss and speech and language developmental delays.^4^ If three or more episodes of AOM occur within 6 months, or four within a year, the infection is diagnosed as recurrent acute OM (RAOM),^5^ or when middle ear fluid persists for 3 months or longer, as chronic OME (COME).^5^ The surgical placement of tympanostomy tubes (TT)^6^ can be used to treat these conditions. As such, TT placement is one of the most common outpatient procedures performed under anesthesia for children.^7^ It has been theorized^8,9^ that middle ear biofilms play a significant role in the persistence of RAOM^10,11^ and COME.^12^ Biofilms are communities of bacteria that have altered genetic expression profiles^13^ and a self-excreted exo-polymeric matrix that promote increased resistance to host immune system activity and antibiotics.^14^ Biofilm-mediated infections in the ear, other regions of the upper respiratory tract, the urinary tract, and on implanted catheters and prosthetics, are all difficult to treat.^15^

To diagnose OM, physicians typically utilize a standard otoscope to visually assess the eardrum (tympanic membrane, TM). Otoscopes provide surface illumination and magnification of the TM and allow for qualitative interpretation of visual indicators related to OM infection, including the coloration and transparency of the TM and presence of middle ear fluid. Pediatricians and otolaryngologists intimately familiar with OM have an estimated diagnostic accuracy in the range of 50–70% using otoscopy,^16–18^ although early career physicians remain unquantified. Given the difficulty in properly diagnosing OM, AOM is frequently misdiagnosed. To effectively treat patients diagnosed with OM, current treatment protocols attempt to provide recommended best practices for antibiotic prescription and TT surgery by integrating evidence-based medicine though a systematic review of past studies and data.^1^ Treatment protocols are designed with this uncertainty in mind, aim to reduce antibiotic over-prescription,^19^ and mitigate antibacterial resistance proliferation.^20^ Despite these efforts, treatment for AOM is one of the most common reasons for children to be prescribed^5^ and even overprescribed^21^ antibiotics, and the distribution of antibiotics remains high.^22^

Other tools, such as tympanometry, acoustic reflectometry, and pneumatic otoscopy, can assess the middle ear using more functional metrics. However, tympanometry^23^ or acoustic reflectometry^24^ are often recommended to be used in conjunction with otoscopy for a more complete assessment of infection status.^5^ Pneumatic otoscopy is recommended as the gold-standard for OM assessment,^1^ and when used properly, has an improved diagnostic sensitivity of 94%.^17^ However, it is difficult to utilize properly and is often not used in practice, perhaps due to a lack of training or challenges in properly sealing the ear canal.^5,25^ Overall, there is an unmet need for a tool and methodology that provides a straightforward and quantitative assessment of middle ear pathology for a consistent and reliable diagnosis of OM.

**Fig. 1:**
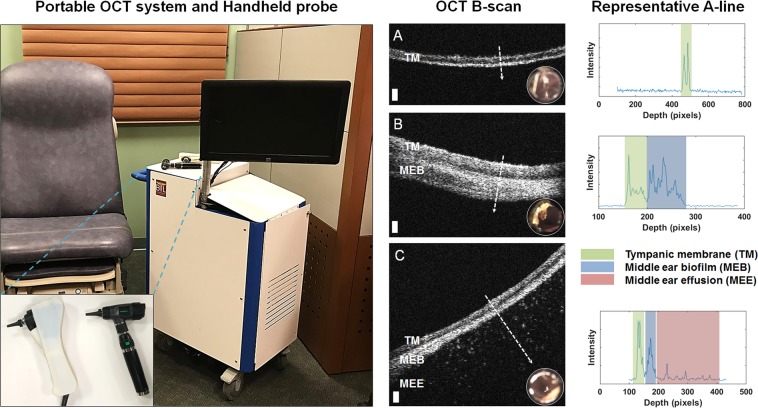
Left: Portable optical coherence tomography (OCT) imaging system and handheld probe. This system was utilized to collect human subject data as part of several past and ongoing clinical observational studies in both outpatient and intraoperative surgical environments. The handheld probe and digital otoscope are shown inset. Right: Representative OCT cross-sectional (B-scan) images and A-line profiles. **A** OCT and digital otoscopy (inset) data from a normal ear. **B** Data from an ear with a middle ear biofilm (MEB). The A-line profile shows additional scattering behind the TM. **C** Subject with middle ear fluid (MEF) and a MEB. The scattering profile shows three distinct regions in the scan. White dashed lines denote the location of the A-line scan within the OCT B-scan. Scale bars represent 100 micrometers in depth

Optical coherence tomography (OCT), a noninvasive cross-sectional imaging technique, is one possible tool that can quantitatively assess the TM and adjacent middle ear space^7,26–29^ for OM. OCT operates using a principle similar to ultrasound imaging, detecting back-reflections of near-infrared light scattered from within tissue, and provides high-spatial-resolution images of tissue at the micrometer scale. Cross-sectional (B-mode) images can be acquired, which consist of multiple adjacent A-lines (depth-scans) assembled as the optical beam is scanned across tissue. The ability of OCT imaging to identify middle ear fluid has been previously demonstrated,^30^ and more recently, biofilms adhered to the TM during chronic or recurrent acute OM have been imaged with OCT.^31^ Figure 1 demonstrates representative cross-sectional OCT B-mode and A-line data from pediatric human subjects with these features, acquired with the OCT system and handheld probe used in this study.

Despite these imaging capabilities, two significant barriers for translation of this technology remain. First, there are no clinical criteria or diagnostic guidelines to assess OCT images of the TM and middle ear for signs of OM. Second, previous studies that have employed OCT for OM assessment have utilized experts familiar with middle ear imaging, OM, and OCT to interpret and correlate OCT data to currently accepted clinical signs and symptoms of OM. Therefore, there is a need for diagnostic criteria to be defined for OCT images of OM, and employed without the regular involvement of an expert reader, particularly as the cost of this technology is reduced and optimized for point-of-care and primary care use by front-line healthcare providers.

Machine learning (ML)-based assessment techniques may provide one solution to these challenges and help to objectively classify and interpret this data. ML approaches are currently in development to supplement radiologist and pathologist diagnostic capabilities for most medical imaging techniques (X-ray,^32^ MRI,^33^ Hematoxylin and Eosin (H&E)-stained pathology,^34^ and ultrasound imaging^35^), following existing standardized diagnostic criteria. Some of these approaches are even beginning to exceed the average radiologist performance, such as one recent algorithm for detecting pneumonia from chest X-ray images.^36^ Guidelines for the identification and classification of disease states with OCT imaging are currently under development for ophthalmology,^37–42^ cardiology,^43^ intravascular imaging,^44^ dermatology,^45^ and other applications,^46,47^ although none currently exist for otolaryngology applications or OM.

This article details results from the development and validation of an automated comprehensive framework, the exploration of the predictive power of various feature subsets, and the performance of several classifiers to identify OM in OCT images. Challenges for clinical translation and avenues for platform improvements are discussed. Finally, guidelines for the minimum performance of an imaging system are explored, specifically for signal-to-noise ratio (SNR) and resolution, to adapt this method to OCT systems used in clinical point-of-care and primary care settings.

## Results

### Classification platform

**Fig. 2:**
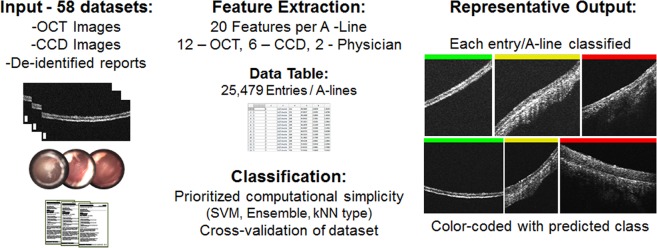
Program overview. Optical coherence tomography (OCT) images, digital otoscopy images, and de-identified patient reports were used to create a database with 25,479 entries. Using this database, cross-validation is performed to train and test several classifier types. Finally, each OCT A-line scan is color-coded with the predicted class after classification (Green = Normal, Yellow = Biofilm, Red = Biofilm and fluid). Representative results shown are correctly classified (100%) and representative of each class

The overall platform operation is shown in Fig. 2. Similarly, clinical findings for subjects in this study are in Supplementary Table 1, with discrepancies between OCT and physician findings bolded. The automated platform first begins feature extraction on the database, which consists of OCT images, digital otoscopy images, and de-identified patient reports. Feature extraction from the dataset of each subject required ~40 s. This generates a large data table to be used for classification. This table is then split into training and test groups to assess performance of the classifier using 58-fold random subsampling cross-validation. A total of 22 classifiers were evaluated to compare the performance of these feature groups, with the performance of the most computationally simple method from each classifier group (Ensemble, SVM, kNN) highlighted in Table 1.

**Table 1:** Performance (accuracy) comparison results between most computationally simple major classifier types in MATLAB, testing eight feature subsets

	Feature subsets	Ensemble	SVM	kNN
		Random forest	Gaussian	Fine
1	Clinical report keywords	82.6	82.6	75.8
2	OMGRADE scale	80.2	80.2	69.0
3	Six digital otoscopy metrics (custom)	96.6	96.6	96.6
4	Physician info (1 + 2)	92.1	92.1	92.1
5	All clinical information (1 + 2 + 3)	100.0	100.0	100.0
6	Twelve optical coherence tomography (OCT) metrics	93.9	90.4	88.9
7	Clinical and OCT features (5 + 6)	100.0	98.4	99.5
8	Least useful 5 removed	100.0	99.6	99.9

### Performance of feature subsets

**Fig. 3:**
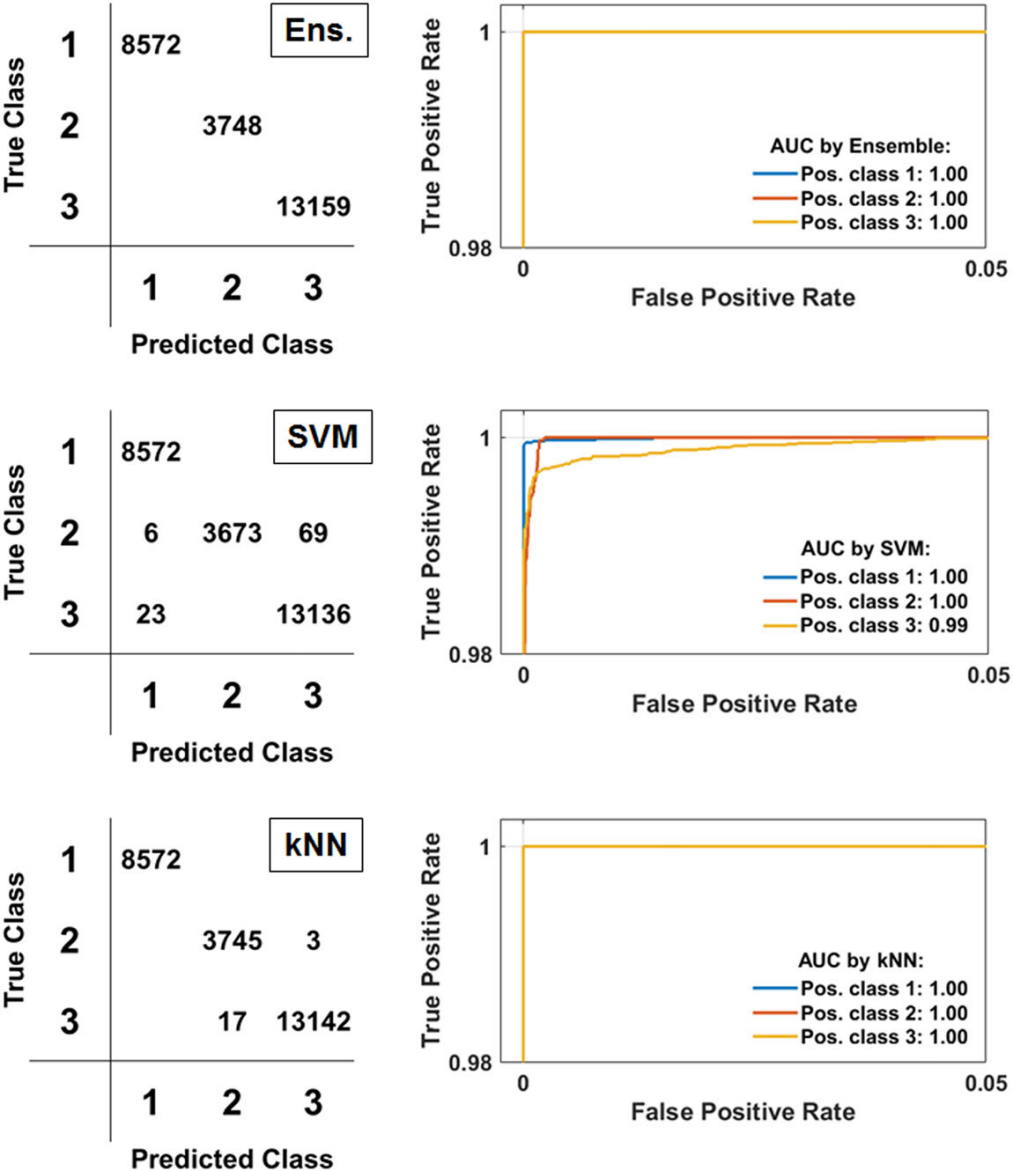
Receiver operating characteristic (ROC) curves and confusion matrices for Subset 8 results. Full testing results from all eight subsets shown in Table 1. Predicted/True/Positive (Pos.) Class 1 = “Normal”, Class 2 = “Biofilm”, Class 3 = “Biofilm and Fluid”. *AUC* Area Under the Curve; *Ens* Ensemble; *SVM* Support Vector Machine; *kNN* k-Nearest Neighbor

Different feature sets explored the utility of different sources of information (from the clinician, OCT, and otoscopy), and which metrics were effective predictors for middle ear infection, as shown in Table 1. Results from Subsets 1 (Clinical reports) and 2 (OMGRADE scale—otoscopy) demonstrate the difficulties of using limited features in a classifier, and perhaps more generally, that a single piece of information is insufficient to make a reliable diagnosis. While achieving reasonable performance on average, each image was broadly classified as either completely “correct” or “incorrect”. The performance of Subset 3 was markedly improved over Subsets 1 and 2. While clinicians do not necessarily have access to these custom created digital otoscopy metrics, they are perhaps indirectly interpreted. When all clinical information is considered together (Subset 4 or 5), improved performance is achieved. Overall, classifier performance in Subset 6 using data from the portable OCT imaging system was improved over Subsets 1–3, in part due to the availability of more than a single feature for classification in each A-line. When all (clinical and OCT) features are used together (Subset 7), improved performance is found with more consistent labeling performance across images, rather than the binary-like labeling in earlier subsets. Subset 8 reduces the feature set by removing the 5 worst performing features as determined in Subset 7 by the out-of-bag error from the ensemble classifier, which reduces computation time by 22% and maintaining roughly equivalent performance. For Subset 8, ROC curves and confusion matrices are provided in Fig. 3.

### Towards a clinically focused application

**Fig. 4:**
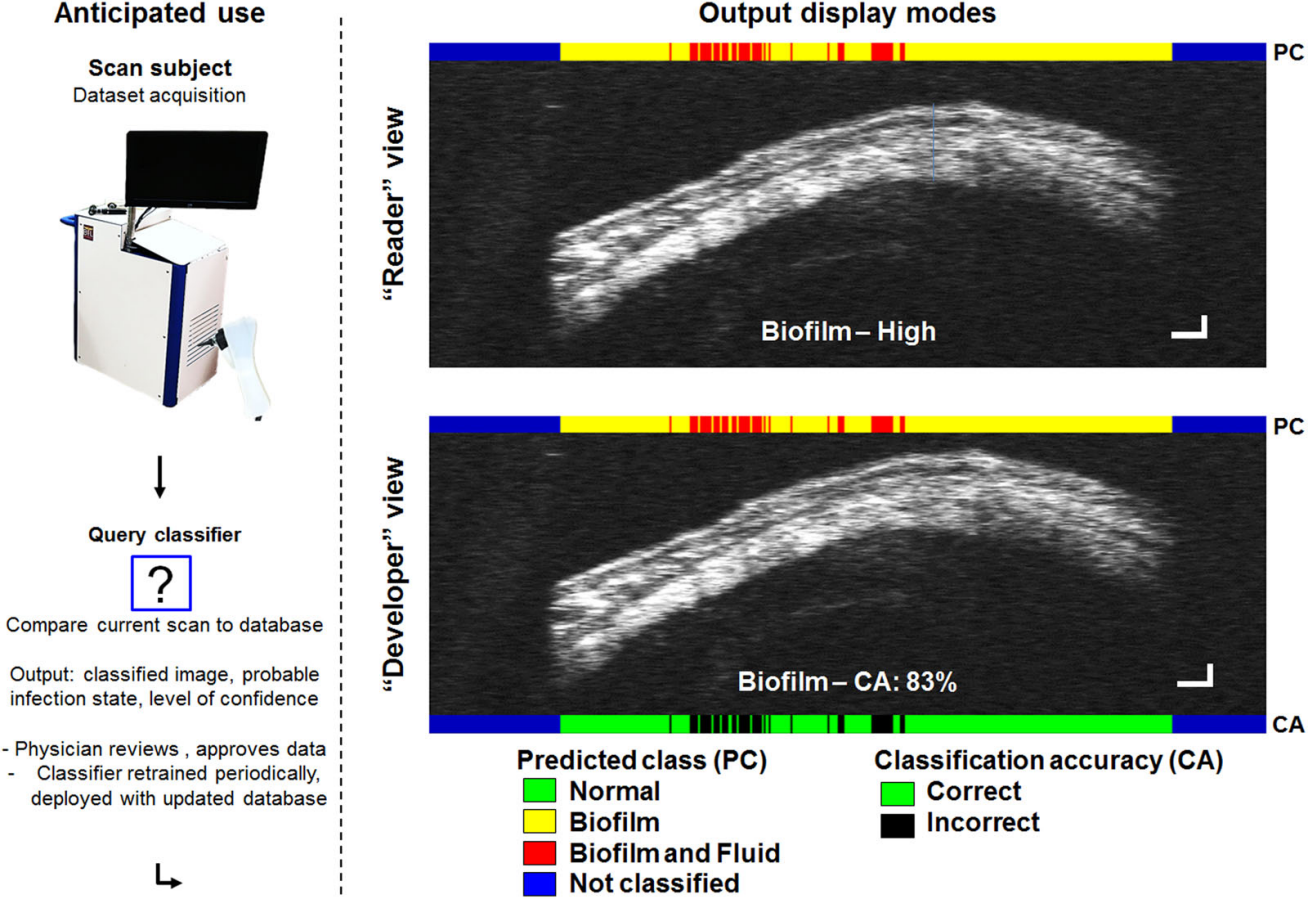
This type of system will be deployed into clinical settings and generate new (previously untrained) datasets, which will be analyzed by the classifier to generate labeled images (right). Two display modes were created to suit expected use cases. “Reader” view (top) is the default output, where the classifier prediction is color-coded at the top of the image (coding information annotated, bottom). The predicted class and confidence (Biofilm–High) can be color-coded, with text, or with both as shown. Images have been widened 3x to demonstrate A-line level granular identification of different regions in the image data. Here, uncropped scans are shown, with empty areas coded in Blue, demonstrating the limited preprocessing steps tolerated by this platform. “Developer” view (bottom) is tailored to assist the development of new features or classifier functionality to identify specific regions within an image. As the class of training data is known, classification accuracy (CA) can be computed and displayed (bottom, 83% of classifier A-line predictions within this image were correct). Scale bars in each dimension correspond to ~100 micrometers. These results can be verified by the physician, “accepted” and integrated into periodic future updates

These results are promising for the utility of the feature extraction method used in this platform for detecting otitis media. However, the real-world usage of this type of system will eventually depend on the classification of new, untested data against a fully trained and deployed classifier. Figure 4 demonstrates this principle and also shows two display modes available in this system, along with the labeling annotations provided for each A-line when recombined into OCT B-mode images (in contrast to labeling the entire image with broad stroke, single output). Uncropped OCT images are shown in contrast to those in Fig. 1, demonstrating the limited preprocessing needed for data used in this platform.

**Fig. 5:**
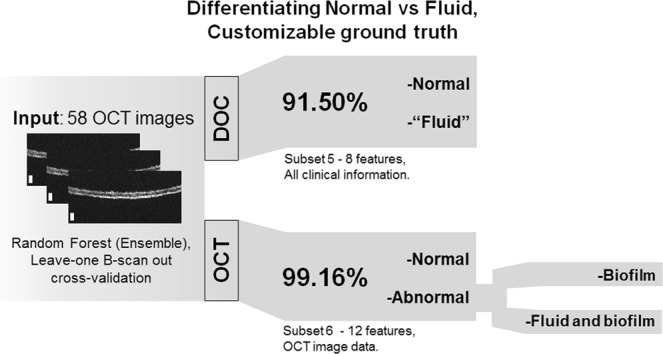
*Can optical coherence tomography* (*OCT*) *discriminate fluid as accurately as a physician?* Using “Leave-one B-scan out” cross-validation and the physician’s diagnosis (DOC) to train the classifier yielded an estimated future accuracy of 91.50%. Using quantitative OCT metrics (OCT) as the ground truth increased accuracy to 99.16% to identify abnormalities in OCT data related to infection. In addition, OCT data can be further utilized to discriminate different types and qualities of infection, including middle ear fluid and biofilms, which is not possible with otoscopy alone

With the development of the platform workflow and initial testing of the classifier pipeline, applied questions and challenges were explored using the more clinically focused set-up. To begin, the platform was first re-tested to ensure it could detect an effusion or middle ear fluid (MEF) using OCT data as accurately as a clinician with otoscopy, the current standard-of-care. Figure 5 provides an overview of the classification conditions and results. This test employed a random forest classifier, as it was the best performer (albeit marginally) in the initial exploration. The physician’s diagnosis using otoscopy was used as the ground truth to identify symptoms of infection using OCT features. An estimated future performance of 91.50% was determined to distinguish normal and abnormal OCT scans with the physician (DOC) as the basis. These results show that using OCT metrics to identify infection is equivalent to a physician’s diagnosis with otoscopy 91.50% of the time. When using the same process, now with the ground truth derived from readers interpreting OCT data for abnormal scans, the predicted future performance increased to 99.16%.

This result suggests that the platform can identify clinically indicated MEF in subjects as accurately as an expert human reader. Interpreting these results, the increased performance may be due to the improved capability of OCT to detect depth-resolved microstructural changes that are indicative of infection, versus visual-only otoscopic observations of surface features, where signs of infection may be missed if subtle, or unintentionally misinterpreted. OCT may therefore be a noninvasive, unbiased, and more effective tool to quantitatively detect signs of MEF and biofilms, compared to otoscopy, especially without the need for an expert human reader when implemented with this automated analysis platform. Apart from identifying the clinical indications of fluid, further differentiation is possible between different types of abnormal scans based on depth-resolved OCT data, now between three groupings: “Normal”, “Biofilm”, or “Fluid and Biofilm”.

Finally, the performance of this leave-one B-scan out method was then tested on the same subsets shown in Table 1. Ultimately, this test achieved 91.57% accuracy using Subset 8. Although this system needs further refinement and additional data to more thoroughly evaluate its full potential, it performed well given the imposed conditions and limited available dataset. Complete results from this alternate testing methodology (using Subsets 1–8) are shown in Supplementary Table 2. In total, these initial classification tests suggest this feature extraction and classification platform may provide one method to potentially improve the clinical diagnosis of OM using OCT, as accurately detecting signs of middle ear infection, including the presence of biofilms and MEF, is crucial to properly diagnose and subsequently treat any patient for OM.^1^

## Discussion

Accurately diagnosing OM infection is a challenging task. Key factors to properly diagnose OM utilizing current national recommended guidelines and practices include accurately identifying the length of time of infection, the determination of any bulging or retraction of the TM,^3,48^ and perhaps most importantly, the presence and type of any MEF.^1^ Children can be mostly asymptomatic with MEF, or OME,^1^ which creates difficulty in establishing an infection timeline. Restless and uncomfortable children can similarly complicate otoscopic assessment and diagnosis.

As previously discussed, available tools to diagnose OM typically provide qualitative information, which may not always suggest a clear diagnostic conclusion. While the otoscope provides visual information about the physical appearance of the ear, distinct infection states are not always observable in daily practice. As the current gold-standard, pneumatic otoscopy can provide additional functional information about the mobility of the TM, but this technique is not often used as previously discussed. Acoustic techniques such as tympanometry and acoustic reflectometry provide additional information about the acoustic response of the ear, but are still only considered to be supplemental tools for a diagnosis of OM. Once a diagnosis is made, physicians must then rapidly determine the best course of treatment, as time in the exam room with each patient can often be limited to no more than 10–20 min^49^ in some clinics. As such, OM is difficult to diagnose, and is one of the leading reasons for children to be prescribed broad-spectrum antibiotics and experience temporary hearing loss.^22^

There are other tools in development that aim to improve the acquisition of specific diagnostic markers used to diagnose OM. MEF can be detected through several means, including gold-standard pneumatic otoscopy, ultrasound,^50^ and a recently developed short-wave infrared wavelength otoscope.^51^ The position of the TM can similarly be detected using light-field imaging.^52^ However, an OCT imaging system integrated within an otoscope as demonstrated here may provide a more complete solution to identify both MEF and biofilms, in addition to many other diagnostically relevant and needed features, including tracking the TM position during deflection by pneumatic modulation.^27^ By providing simultaneous high-resolution, depth-resolved, and quantitative structural, functional, and optical characterization of tissue and MEF, OCT imaging can be performed on tissue without any preparation and with subjects in any state (awake, sedated, or anesthetized).

Currently, there is no accepted method to identify the presence of middle ear biofilms (MEBs), although it is likely that biofilms increase the opacity of the TM during infection. In this study, the development of the “Normal”, “Biofilm”, and “Fluid and Biofilm” states was made possible by observing the image-based features in OCT data in this and past studies. It was observed that subjects with more severe cases of OM have MEF in addition to an accompanying MEB. This raises additional questions about the pathogenesis of MEB during OM; questions that are beyond the scope of this present study.

OCT, however, could be one tool that provides a quantitative identification of biofilms and fluid, and in addition, provide further characterization of the purulence or scattering of the fluid. In this and prior studies, it is common to identify a biofilm layer and middle ear fluid in subjects with more severe cases of OM. As infections progress, any MEF becomes more purulent and optically scattering, depending on the duration of the infection. This is likely due to increasing amounts of immune cell activity and biofilm dispersal within the MEC.^48^ Clinicians do not currently diagnose or treat middle ear biofilms as there is no accepted diagnostic tool, nor established or tested/verified treatment regimen. With these limitations in mind, this platform may offer the immediate potential to identify the presence of MEF and MEB, as well as enable new and expanded capabilities in the future.

The use of ML analysis to classify OCT images from subjects with OM can provide a means to automatically classify data and provide a probable diagnostic outcome. When an image is successfully collected, a combined OCT + ML platform could ensure the user would have a minimum baseline skill for detecting diagnostic markers for OM. In its current form, this platform is intended to supplement the assessment of the numerous quantitative details within the data and apparent in tissue, and integrate statistical measures to help guide decision making. In turn, with an accurate diagnosis, it may then be possible to provide the most appropriate and effective treatment for the current state of infection. This platform is not intended to replace clinical expertize, but offers the potential for further research and clinical investigations before being validated as an approved technology for clinical decision making.

The clinically focused set-up utilizing an RF classifier employed a “leave-one B-scan or subject-out” strategy, with *K* = *N* = 58-fold cross-validation. The classifier is discarded after each training iteration rather than continuously being retrained on all data, and recreated for the next iteration. This strategy attempts to more accurately simulate expected future use and performance of this platform, where an untrained and unknown patient or dataset will be investigated using a pre-trained classifier. The differences in the use of data during classifier training may explain why the MATLAB classifiers perform differently than the custom RF classifier, although this method may give a sense of a lower bound on performance within this current dataset. Different feature sets were also used to test the performance of features extracted from OCT data and clinical data. The best performance was found when a combination of features were used, indicating that both OCT and currently utilized clinical information together provide advantageous and complementary classification information.

In the future, additional features can be added to this platform to improve its versatility and robustness. For instance, data collection can be improved to allow for the determination of the viscosity of in vivo MEF with OCT, which may allow for further discrimination of serous and mucoid fluid types. To ensure usability for real-time analysis, algorithms that ensure rejection of unintended or unwanted imaging artifacts and reflections can be implemented, along with a notification system to request the user to retake data with identified artifacts. Existing metrics and features can also be improved, such as expanding digital otoscopy analysis to include other metrics related to TM coloration, transparency, or opacity.^53^ The presentation of data can also be scaled to suit the expected use. In clinics with technicians or situations where a simpler screening or evaluation is needed, perhaps a simple output can be designed for notification: ‘normal’’, ‘recommend for clinical evaluation’’, or ‘retake data.’’ In a more traditional clinical setting, some physicians may desire to see an expanded set of relevant information, which can be set to include the full image data as well as the metrics described above, as needed.

There are several points in this study that merit further discussion. The three class output labels that were used in this platform take into consideration immediately useful clinical information. In the future, many more infection states of OM and diseases or conditions of the TM can be added, such as TM perforations,^54^ dimeric TMs from previous surgical interventions,^55^ cholesteatoma,^56,57^ or myringo-/tympanosclerosis.^55^ This platform can readily expand to accommodate these additional states, although additional testing with appropriate and sufficient training data for each newly added condition will be required to assess accuracy. Examples of a dimeric TM and a TM with myringosclerosis are shown in Supplementary Fig. 1. While OCT imaging can identify biofilms, currently there is no recommended course of action for treatment of a biofilm within current guidelines, nor is there any clinically accepted diagnostic method to noninvasively identify biofilms in the middle ear.^58^ As biofilm-related infections are persistent due to their innate ability to resist the host immune response and antibiotic treatment,^59,60^ it is expected that management strategies for chronic OM will follow treatment strategies used for other biofilm-mediated infections, such as cystic fibrosis^61^ or other respiratory infections.^62^ Finally, our group is currently developing systems with reduced off-the-shelf costs,^28,63^ most recently culminating in a portable briefcase form-factor.^64^ Such systems are portable and can be easily transported to non-traditional point-of-care settings, and are suitable for cost-averse clinical disciplines like primary care.^64^ Suggestions for implementing this system in different and/or lower-cost platforms are included in the Methods section.

## Methods

### OCT system and human subject imaging

A previously developed custom-designed portable and handheld OCT system, shown in Fig. 1, was used to image human subjects and characterize various presentations of OM in clinical studies.^27,29^ Briefly, this system is based on a spectral-domain OCT engine with a center wavelength of 860 nm and an approximate bandwidth of 135 nm. The system has an axial resolution of 2.4 μm and a lateral resolution of 15 μm. The system emits an optical power of 2.5 mW onto the tissue, which is well below the ANSI standard safety limits for incident exposure. A digital otoscope (Welch Allyn, USA) was used to acquire digital otoscopy images of the TM. Further details can be found in an earlier publication.^30^

Pediatric subjects were recruited and observed under IRB (Institutional Review Board)-approved protocols (approved by both Carle Foundation Hospital and The University of Illinois at Urbana-Champaign) at the primary care/physician’s office, specialist otolaryngology clinic, or intraoperatively in the surgical ward at Carle Foundation Hospital, Urbana, IL. Informed consent was acquired from all participants by hospital research staff. For this study, 58 previously imaged subject datasets were selected from an internal data repository. Each dataset consisted of a representative cross-sectional OCT B-scan image, a color digital otoscopy image, and a de-identified clinical subject report. These subjects reflect the range of patient presentations of OM, consisting of healthy normal controls, and subjects diagnosed with acute otitis media (AOM), otitis media with effusion (OME), chronic otitis media with effusion (COME),^1^ and recurrent acute otitis media (RAOM).^3^ Clinical findings from each dataset are shown in Supplementary Table 1, including the clinical impression of the presence of fluid, as determined by a physician’s assessment using otoscopy (OTO), and a reader examining OCT data (OCT). Discrepancies in the presence of middle ear fluid (MEF) between these two analysis methods are bolded.

### OCT image groupings and reader study

To interpret and label OCT training data appropriately, a small blinded reader study was performed. Three readers familiar with OCT, OM, and middle ear imaging were trained through a guided analysis of two representative sample images from each group, consisting of data not used in this study. Then, each reader evaluated and classified each OCT image in this dataset into one of the group classifications used in this study (“Normal”, “Biofilm”, “Biofilm, and Fluid”). Trends identified in OCT images were used to develop these groupings, as no currently accepted noninvasive clinical techniques provided information related to the presence of middle ear biofilms.^58^ These groupings were developed from our past observational clinical studies where OCT images of subjects with OM were correlated with clinical findings reported by physicians, or where intraoperative OCT imaging was directly compared to surgical intervention and microscopy findings during TT surgery. Normal cases were identified by a TM of ~100 micrometers thick and the lack of any additional structures (biofilm or effusion) observed in infection states. The second grouping (“Biofilm”) was created as otoscopy does not provide an indication of the presence of a middle ear biofilm, perhaps only the appearance of a dull, thickened, or opaque TM. The third grouping (“Biofilm and Fluid”) was created due to the presence of a biofilm in all scans that contained MEF. These classes are shown in Fig. 1. Further discussion of these correlative studies and results are described in previous publications.^26–31,65–69^

The consensus or majority vote of the three readers was used as the final label for this classification. Overall, there was little variation in reader assessment. For 58/58 scans, at least 2/3 readers agreed on the group label, with all readers completely agreeing on 38/58 subjects.

### Classifier set-up (1/3): defining and extracting features

Features were developed to extract or capture inherent qualities of tissue, utilizing a broad range of physical/structural, clinical, or optical metric to numerically quantify infection states. A total of 20 features (briefly enumerated in Supplementary Table 3) were extracted from each dataset, with 12 unique to each OCT depth profile, 2 derived from the physician’s clinical assessment of each subject, and 6 from digital otoscopy images specific to each subject’s ear.

OCT-based features aim to quantify information about the optical properties of the tissue, which are known to vary with the disease-state of the ear. An automated extraction subroutine first identifies areas of interest in each OCT scan with no preprocessing of data directly gathered from the imaging system shown in Fig. 1. Then, a peak-finding algorithm and various statistical measures quantified the density and distribution of tissue based on the location and distribution of scatterers in each depth scan. To avoid erroneous peaks, noise-floor thresholds were set dynamically on each depth scan using Otsu’s method.^70^ Additional metrics such as attenuation and optical thickness were similarly calculated. When comparing infection groups, this information can help differentiate the physical dimension, amount, and type of tissue when comparing infection states with varying amounts of solid tissue, fluid, and biofilms.

All OCT images were first median filtered using a 2 × 2 kernel and window averaged by a 5-pixel lateral window to reduce noise or speckle, and increase uniformity for analysis. Next, the image area was automatically segmented, and a depth profile of each position was isolated, which was taken radially from the TM surface into the MEC. This process is discussed in detail in a following section. The following features were extracted from OCT data:

#### Optical thickness

The optical thickness (extracted radially) of the TM and any associated MEB and/or MEF has been shown to be statistically linked to infection state.^26^ MEF, bacterial components, or MEB are present across varying infection states, and are not present in healthy controls.

#### Standard deviation of peak-position location

The distribution of scatterers in depth relates to the amount of tissue detected by the OCT system, and the relative distribution in depth. In healthy ears, only a limited number of peaks will be identified within a 100 micrometer range (typical thickness of a human TM), whereas in cases with an effusion or biofilm present, this value will be larger.

#### Mean, standard deviation of peak width

Peak width statistics refer to the size and distribution of the scatterers, as with OCT it has been observed that MEF typically becomes more purulent as an OM infection progresses. The mean peak width correlates to the average physical size of scatterers in a scan. The standard deviation of peak width correlates to the distribution of physical sizes of scatterers in a scan.

#### Mean, standard deviation of peak prominence

Peak prominence statistics relate to the optical composition and distribution of scatterers, as interfaces with larger local refractive index differences give rise to higher backscatter intensity OCT signals. During OM infection, the TM becomes inflamed, with an influx of interstitial fluid and blood,^26^ which have different optical properties to that of bacteria, mucous, and scatterers within an effusion. Mean peak prominence correlates to the different optical properties of scatterers, compared to the surrounding medium. The standard deviation of peak prominence correlates to the distribution of the varying optical composition of tissue in a single scan.

#### Total number of peaks

The total number of peaks is correlated to the density of the tissue or media, and increases with the presence of fluid or biofilm during an infection. Scattering distributions or profiles of normal and abnormal cases have been detailed in previous publications,^27,68,71^ or as shown in Fig. 1.

#### Optical attenuation maximum

OCT provides depth-resolved quantitative structural and functional information. The optical attenuation can be calculated by utilizing a previously developed method that calculates the depth-wise attenuation coefficient at each pixel^72^:

1 $$\mu [i] \approx \frac{{I[i]}}{{2{\mathrm{\Delta }}\mathop {\sum}\nolimits_{i + 1}^\infty {I[i]} }},$$

where ∆ is the (depth) pixel size, and *I*[*i*] is the intensity value at a given depth location *i*. This formula was applied over the previously fitted region of interest identified using the radial fitting from Feature #1 (Optical thickness) to ensure it is calculated over valid points. This method provides numerical discrimination of the different scattering properties of different tissue types. The maximum attenuation in a single depth scan may differ between infection groups, related to the properties of differential components in healthy ears and in cases of infection.

#### Optical attenuation mean, sum over peak-detected depth

The mean attenuation differential between infection groups will differ based on the additional presence of MEF and biofilm components. The sum will provide a measure of the overall signal attenuation in the depth scan. Scans with additional biomass are expected to have higher attenuation than scans from a healthy subject.

#### Fourier width, peak prominence of central peak

Fourier analysis of OCT A-line data provides information regarding periodic or structured features in tissue, represented numerically by analysis of each peak width and prominence. The width of the central peak provides differential frequency-based information related to the optical properties or size of present structures, such as sparse scatterers within a fluid (high frequency) or larger structures such as a biofilm or dense fluid (low frequency). The prominence of different scans correlates to the ratio of low and medium frequency terms in an image, related to the optical properties of tissue.

Additional features were derived from patient assessment clinical reports and digital otoscopy images. In this study, physicians first performed a physical exam on each patient to assess their overall state of infection and prevent any bias in final diagnosis. Clinical reports were parsed for keywords (shown in Supplementary Table 4) related to otitis media or the health of the ear. For the purposes of this study, digital otoscopy metrics were collected to assess the TM for infection, which was converted and quantified using the OMGRADE scale.^53^

#### Otoscopy graded score/OMGRADE

Otoscopy images were analyzed using the OMGRADE scale,^53^ which provides a numeric score for grading the state of infection of the middle ear based on features identified with otoscopy. This scale ranges from 0–6 to distinguish different pathological conditions. Briefly, Grade 0 is a transparent TM in normal position, Grade 1 shows an identifiable fluid level, Grade 2 is an opaque identifiable fluid level in the MEC, while Grade 3 is a completely opaque TM although in a normal position. Grade 4 is a completely opaque and bulging TM, Grade 5 is an opaque TM with bullous formations or a contourless TM with swollen patches of keratin. Grade 6 corresponds to the presence of a perforation in the TM, retraction pocket, or cholesteotoma with or without discharge.

#### Physician’s report score

Physician’s reports are vital to properly correlate image-based features with clinical symptoms. Available reports were parsed for keywords^73^ that provide some indication of a healthy control or instances of OM and related risk factors. Each keyword was given a numerical value and an overall score was tabulated for each subject. Cues related to normal healthy controls or from OM-unrelated visits to the physician were awarded 0 points, such as “unremarkable ears” or “clear TM”. Keywords assigned 1 point include “inflammation”, “effusion”, “erythema”, “inflamed”, “smoke”, “family history of OM.” Two (2) points were awarded to keywords such as “antibiotics”, “referral”, “persistent”, or “purulent.” A complete list of the terms used for scoring is shown in Supplementary Table 4. Higher scores related to more advanced infections. While this metric is empirical and specific to the language used in these reports, the composite score represents the clinical findings of the physicians involved in this study, and by extension, the inherent difficulty in diagnosing OM. Other risk factors,^73^ such as the time of year of the report, age of the subject, and audiological exams (if available), were considered in this scoring system, but ultimately not included due to the complexity in assigning a score to multi-factorial data.

Finally, six metrics from digital otoscopy^74,75^ were developed to discriminate normal and abnormal tissue given different color profiles of the TM typically observed as part of the physical exam. Although these values are not directly reviewed by physicians, this was an additional method to quantify the exam process. Digital otoscopy images were collected using a digital otoscope tool, which ensured consistent illumination and sensor performance between imaging sessions. Earwax, which is a confounding factor and unrelated to infection state, was manually segmented out. Images were then converted to Hue, Saturation, and Luminance (HSL) color space to separate, extract, and quantify color (hue) and saturation separately from illumination information.

#### Hue: average, median, median absolute distance value across otoscopy image

The values calculated from the hue of the image relate to the color of overall redness, injection, or erythema from the surface of the TM. The average value of hue across an otoscopy image was related to the average color shade across the image of the TM. The median value of hue provides differential information from the average, especially in cases where the TM coloration is skewed (non-uniform) across the image. The median absolute distance provides a measure of spread, statistical dispersion, or the width of the distribution of color shades in a single image.

#### Saturation: average, median, median absolute distance value across otoscopy image

The values calculated from the saturation of the image relate to the intensity of the color of overall redness, injection, or erythema from the surface of the TM. The average value of saturation provided another measure of the intensity of color, which relates to infection state. The median value of saturation provides another related measure of the uniformity of the intensity of color. The median absolute distance provides a measure of spread, statistical dispersion, or the width of the distribution of the intensity of color in a single image.

With these 20 features defined, each extracted depth profile had 20 quantitative values calculated and placed in the corresponding database entry.

### Classifier set-up (2/3): ground truth, features assessment

For each classification experiment, several major elements were selected: the classification method, the feature subset used in the classifier, and the ground truths assigned to the features that distinguished the groups.

#### Classifier method

Twenty-two classifiers (available at the time of writing) within the classification learner app in MATLAB were utilized to initially classify the dataset, consisting of SVM, kNN, and ensemble techniques. The most computationally simple method from each group was selected to compare performance, and was implemented using the suggested initial settings in the program, briefly: kNN (“Fine”, 1-neighbor, Euclidian distance, equal weighting), SVM (“Gaussian” kernel, 1v1 multi-class), and Ensemble (“Bagged Trees”, 30 learners). Other tests utilized a random forest (RF) classifier^76,77^ to test a more clinically focused classification set-up, where instead of randomly subsampled training and test sets, a leave-one B-scan out technique was selected.^44,47^ In part, this combination was utilized to investigate whether this would reduce error in unbalanced datasets where data may be limited. In addition, to avoid sensitivity to incomplete data within a specific data vector, such as missing otoscopy images or physician’s notes, and can even rank the most useful features for classification. Briefly, this strategy works by splitting N total images into a training set (N-1 images) and setting aside one image for testing. Each image in the dataset was tested on the trained classifier, and the mean accuracy was calculated across all loops or “folds”, which served to estimate the expected future performance on untrained data.

#### Feature subsets

Different feature subsets were utilized to compare the predictive ability of information gained from different parts of the clinical examination. If certain features are chosen, the resultant classification performance can help determine, which features are most relevant to identify signs of OM in OCT images. In the Results section, the ability of otoscopy and OCT to identify the presence of fluid in subjects was tested with this platform, among other comparisons of interest.

#### Ground truth

To begin, the data was sorted and labeled to accurately reflect the clinical indications of the subject and the corresponding OCT metrics. The absolute ground truth for diagnosing OM is invasive surgical evaluation of the middle ear contents of each subject. However, when considering typical examination methods, invasive surgical inspection is impractical to perform given limitations of time and resources in daily practice. For this test, the ground truth basis was either derived from the physician’s impression using otoscopy as stated in the clinical reports (DOC), or from the consensus of three readers analyzing OCT images (OCT) as previously described. Comparing different ground truths in this manner allowed a direct comparison between the capability of the current “gold-standard” and OCT as a new technique.

### Classifier set-up (3/3): displaying and presenting data

The presentation of the results from this framework was developed to be relevant for clinical applications as demonstrated in Fig. 4. Two viewing modes, “Reader View” and “Developer View” were implemented. The “Reader view” is the default output, which annotates the OCT image with the predicted class of each depth profile and allows for quick visual interpretation of classifier results. The class is color-coded to assist in quick discrimination of infection state, with “Normal” cases in green, “Biofilm” in yellow, and “Fluid and biofilm” in red. The expected class of the image and expected accuracy are displayed, ranging from High (>80%), Medium (>50%), Low (>20%), and error. In “Developer View”, the exact numerical classification accuracy is shown, which compares the expected class to the provided group labels in the training set data. This mode was useful when modifying feature detection, segmentation, or adding new features, to ensure proper functionality and performance. In principle, the complexity of the display modality can be adjusted to suit any range of needs. For example, it could display simply an error/green/yellow/red light, indicating the severity of infection or need for referral, or to retake a scan.

### Computational hardware

All results from this work were computed using an off-the-shelf PC with a Core i7-5960X3 GHz CPU, 32 GB 2666 MHz RAM, a 7200 RPM HDD, and a GTX 1080, although the GPU was not used for processing or analysis.

### System requirements and suggestions for data collection

To abstract the use of this platform with any comparable system, guidelines for OCT images and otoscopy images were defined and evaluated. In general, all OCT data should be of similar quality in regards to axial and spatial resolution, SNR, wavelength, and other imaging properties, such that comparisons can be made of the underlying physical features that are being detected. While B-scans are advantageous to utilize information from adjacent A-lines, such as for window averaging, fitting, and analysis as described above, it is not strictly required as this system ultimately relies on A-line data to classify these infectious states.

Additional pre-processing steps can be undertaken that help to reduce the discrepancies when using multiple systems to collect data that feed into a common database, such as cropping images to remove artifacts in OCT, segmenting data to remove unrelated image features such as earwax, or by reducing illumination variances in otoscopy. OCT data must be free of wrapping, back-reflections, saturations, scanning or other optical artifacts. For consistency, images should be taken and compared from the same region of the ear. The light-reflex region is easily identifiable in most subjects and was chosen as the physical landmark on the TM for images in this study.

While the intensity values in the OCT data are not used for classification, the SNR of an OCT system image must be of sufficient quality to clearly resolve tissue features throughout the imaging window, and also ensure that the image SNR is of sufficient quality for later analysis metrics to be successfully detected and computed. This was determined empirically by taking high-quality scans from the currently used system and later synthetically degrading their quality using additive randomized Gaussian speckle noise. These degraded images were fed back into the classifier to observe performance degradation. SNR was calculated using a 20 pixel square region of interest for both signal and background from a set of representative OCT images (Eq. 2). Current data empirically suggests that 55 dB is the approximate lower limit before fitting and classification performance begins to seriously degrade as demonstrated in Supplementary Fig. 2, while ~80 dB and above is optimal.

2 $${\mathrm{SNR}}\,({\mathrm{db}}) = 20\log _{10}\left( {\frac{{I_{{\mathrm{Signal}}\,{\mathrm{ROI,mean}}}}}{{\sigma _{{\mathrm{Background}}\,{\mathrm{ROI}}}}}} \right)$$

A minimum resolution for the OCT system is difficult to define, as the features of interest for classification must be detected and discriminated between each classification group, such as sparsely scattering MEF or thin biofilm structures. Still, in the current implementation, it was again empirically determined when degrading OCT image resolution using a Gaussian blur function that the resolution can be as low as 12.5% of the original system resolution (effectively 19.2 μm, originally 2.4 μm) and still achieve adequate classification results, although overestimating the severity of present features due to the reduced resolution. This is visually demonstrated in Supplementary Fig. 3. However, as more data and classification groups are added for more complex pathology, higher resolution is typically always beneficial.

Digital otoscopy images should show as much of the TM under sufficient yet non-saturating illumination as possible, ensuring the same lighting spectrum is used (cool, warm, etc.) across imaging sessions and subjects. In practice any otoscope or surgical microscope image could be used. Quantitatively, the saturation value (S channel in the HSL color scale) should be >10% but <95% to ensure accurate color detection. These values ensure the color spectrum does not contain dim and gray (lower bound), or overly saturated (upper bound) data. Standard-of-care protocols do not call for the removal of earwax unless it significantly impedes assessment of the TM and middle ear.^78^ Still, the physical removal of earwax is suggested, if possible, to collect higher quality digital otoscopy images. Software-based algorithms are being developed that can alleviate this need and segment out earwax from images,^74,79^ although complete blockages do sometimes occur and require removal.

### Radial OCT depth profile extraction

Axial thickness of ear structures for each A-line was initially calculated by finding the distance between the first and last peak in the current A-line position (Eq. 4).

3 $${\mathrm{Peak}}\,{\mathrm{positions}}\,{\mathrm{at}}:(x_m,y_{n,{\mathrm{Top}}}){\mathrm{,and}}\,(x_m,y_{n,{\mathrm{Bot}}})$$

4 $${\mathrm{Axial}}\,{\mathrm{distance}} = y_{n,{\mathrm{Top}}} - y_{n,{\mathrm{Bot}}}$$

Although straightforward to implement, axial thickness does not always accurately quantify a naturally curved TM, shown in Supplementary Fig. 4A, which depending on the scan geometry of the subject and handheld OCT probe, could also be angled within the OCT image. As B-mode OCT images were available, an alternate method was developed.

Radial thickness takes advantage of information from adjacent A-lines in an OCT B-scan to detect and calculate thickness through a point normal to the tissue surface. Each A-line is now run through the peak-finding algorithm (using the 50 pixel threshold as before) to find the top edge of the tissue using the first peak, where a point is added to a mask image in the same location if it is within a separation window, described below. As each A-line is processed, a point-cloud like mask is generated for the top line. The final outline of a representative scan is shown in Supplementary Fig. 4B.

As this algorithm traverses each A-line, each point must be within a fixed separation window from the previously identified point:

5 $$(x_m,y_n) \to (x_{m + 1},y_{n + 1}),\Delta y \,<\, {{\mathrm{Separation}}}\,{\mathrm{Window}}$$

The surface of the TM is relatively flat when comparing adjacent pixels, even after window averaging. Adjacent points that vary wildly are likely due to a misidentification in the peak-finding algorithm. If no peak is detected, or a peak is detected outside this separation window, it is discarded and the separation window on the subsequent iteration dynamically increases until a new peak is found. This behavior prevents failure from occurring when processing slightly obscured regions of tissue, discontinuities in tissue surfaces, or in regions of low SNR, which often occur during imaging if the ear canal or earwax partially obscures the cross-sectional OCT beam. Once a point is eventually found on a subsequent iteration, the separation window is reset to its original value. Once the top point mask is generated, the mask is thresholded using the original intensity image to reduce the effect of outlier points, and a 4^th^ order polynomial is fitted to this line (Eq. 6), which tries to find the average path through these points, and is set aside as *y*_Top_.

6 $$y_{{\mathrm{Top}}/{\mathrm{Bot}}} = Ax^4 + Bx^3 + Cx^2 + Dx + E$$

The bottom point mask and fitted curve is similarly created (Eq. 6), instead using the last detected peak, and using a separation window with a starting value three times greater than the top curve. This allows for proper detection of points in deeper areas of the image where sparse scatterers, detector roll-off, and low SNR play a larger role than near the zero-delay or top of the image in the OCT system. Functionally, this ensures areas of low-scattering fluid in OCT images are more likely to be detected. The fitted curves for both sets of points is shown in Supplementary Fig. 4C.

Once these two polynomial curves are generated, the radial thickness of each A-line is calculated by translating across each point on the top line, using these known points (*x*_1:*m*,Top_, *y*_1:*n*,Top_) to find a solution on the bottom curve. This occurs in one of two ways depending on the curvature local to each point. First, a normal line from the top curve is calculated (Eqs. 7 and 8), and intersection point(s) with the bottom curve are identified if possible (Eq. 9). The radial thickness (Eq. 10) ensures that the thickness of this region of tissue is more accurately mapped. Empirically, this has been found to be more accurate and have a lower standard deviation across the tissue than the axial thickness, as shown in Supplementary Fig. 4D. While each point on the top curve has a solution on the bottom curve, the points demonstrating fitting in Supplementary Fig. 4C, D have been downsampled for display purposes.

7 $$m_{{\mathrm{NormTop}}} = - \left( {\frac{{\partial (y_{{\mathrm{Top}}})}}{{\partial x}}} \right)^{ - 1}$$

8 $$y_{{\mathrm{NormTop}}} - y_{n,{\mathrm{Top}}} = m_{{\mathrm{NormTop}}}(x - x_{m,{\mathrm{Top}}})$$

9 $${\mathrm{Solve}} \to (x_{{\mathrm{solution}},{\mathrm{Bot}}},y_{{\mathrm{solution}},{\mathrm{Bot}}});y_{{\mathrm{NormTop}}} - y_{{\mathrm{Bot}}} = 0$$

10 $${\mathrm{Radial}}\,{\mathrm{distance}} = \sqrt {(x_{m,{\mathrm{Top}}} - x_{{\mathrm{solution}},{\mathrm{Bot}}})^2 + (y_{n,{\mathrm{Top}}} - y_{{\mathrm{solution}},{\mathrm{Bot}}})^2}$$

If no solution exists within at the current position along the top line, a second method is employed to find the closest point on the bottom curve. Typically, this occurs when the point of analysis on the top line is near the edge of the imaging window, and a normal line from the top curve doesnot have sufficient space to intersect with the bottom curve. In this case, the shortest distance is found from the analysis point to the bottom curve (Eqs. 11 and 12).

11 $${\mathrm{dist}} = \sqrt {(x - x_m)^2 + (y_{{\mathrm{Bot}}} - y_n)^2}$$

12 $${\mathrm{Solve}} \to (x_{{\mathrm{solution}},{\mathrm{Bot}}},y_{{\mathrm{solution}},{\mathrm{Bot}}});\frac{{\partial ({\mathrm{dist}})}}{{\partial x}} = 0$$

The distance to the analysis point is then computed (see Eq. 11), and is compared against both the equivalent axial thickness value for this same position (see Eq. 4) and the thickness value from the previous iteration dist((*x*_*m*,Top_, *y*_*n*,Top_) → (*x*_solution−1,Bot_, *y*_solution−1,Bot_)) if it exists. The minimum of these three values is then selected. A final check analyzes slope of the top line. If it is near 0, this indicates the analysis point is near a peak or valley and ensures the axial thickness is used.

This second part of the fitting process prevents some A-lines, typically near the edge of the image, from fitting to image data that would stretch past the edge of known data, and instead locks to the corner point on the bottom line. However, this may lead to an improper characterization of thickness. While the function of the lines that define the segmented area could still be calculated past the image boundaries, the points in these regions are undefined and could lead to significant errors. A comparison of these methods is shown in Supplementary Fig. 5. Ignoring these edge points reduces the overall dataset size by 20% (from 25,479 to 20,327 entries overall) and does not change the measured thickness values significantly. However, loss of data in this limited database will detrimentally impact short-term future performance, especially considering the overall accuracy of this platform was not significantly improved from this change. As shown in the plot in Supplementary Fig. 5, the data with edge A-lines removed from processing (Orange line) only slightly reduces overall accuracy when compared against all data (Blue line). As the current dataset is limited, including as many data points is of immediate interest to ensure the flexibility and stability of this system. With additional data added to this classifier database over time, edge case A-lines can eventually be safely removed without much loss of performance or accuracy in the future.

## Supplementary information

Supplementary material.

## Data Availability

Data and materials used in this study are available upon reasonable request to the corresponding author and under a collaboration agreement.

## Supplementary information

**Supplementary information** accompanies the paper on the *npj Digital Medicine* website (10.1038/s41746-019-0094-0).

## Code availability

Computer code used in this study is available upon reasonable request to the corresponding author and under a collaboration agreement.
