# Supplementary material for: Automated classification platform for the identification of otitis media using optical coherence tomography
Source: NPJ Digit Med. 2019 Mar 28;2:22. doi: 10.1038/s41746-019-0094-0 (PMC6550205; doi:10.1038/s41746-019-0094-0)
Supplement: Supplementary file 1 — Supplementary material. [file 41746_2019_94_MOESM1_ESM.pdf]

Automated classification platform for the identification of otitis media using optical coherence tomography (Supplementary material)

Guillermo L. Monroy[1,2], Jungeun Won[1,2], Roshan Dsouza[2], Paritosh Pande[2], Malcolm C. Hill[3,4], Ryan G. Porter[3,4], Michael A. Novak[3,4], Darold R. Spillman[2], Stephen A. Boppart*[1-5]

[1]Department of Bioengineering, University of Illinois at Urbana-Champaign, Urbana, IL
[2]Beckman Institute for Advanced Science and Technology, Urbana, IL
[3]Carle Foundation Hospital, Otolaryngology, Urbana, IL
[4]Carle Illinois College of Medicine, Urbana, IL
[5]Department of Electrical and Computer Engineering, University of Illinois at Urbana-Champaign, Urbana, IL
*Corresponding author

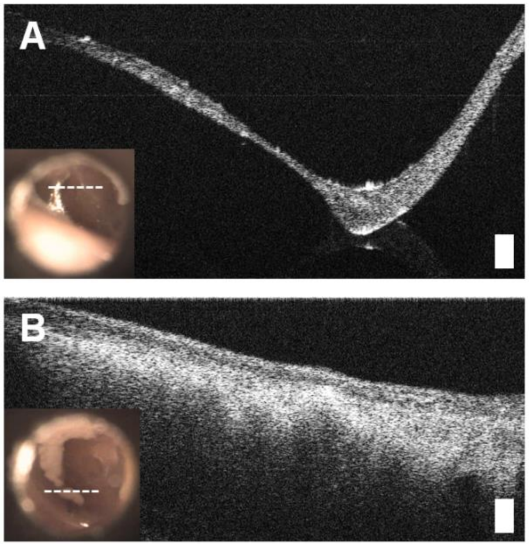

Supplementary Figure 1: OCT images demonstrating infrequently observed pathology of the TM. A: A dimeric TM that shows thinning of the fibrous layer. B: A TM with myringosclerosis. There are several conditions unrelated to an immediate OM infection but occasionally encountered in clinical practice. Datasets like these may confound the classifier in its current form due to segmentation issues in thinned regions, or misclassification of myringosclerosis as biofilms and fluid. With additional training data, cases outside of the expected OM-related class groupings can be managed. Scale bars indicate 100 micrometers in depth.

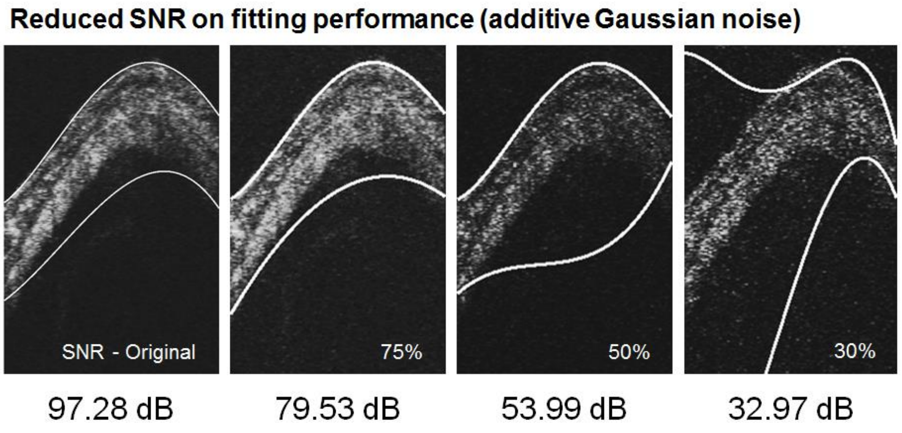

Supplementary Figure 2: Synthetically decreased signal-to-noise ratio (SNR) and fitting performance. Using additive Gaussian white noise, the SNR of test images (inset in each panel) was synthetically decreased and classified to empirically determine estimated SNR requirements for this platform. Fitting performance severely degrades near 33 dB (30% of original), leaving 55 dB (50% of original) as the approximate lower limit, and 80 dB (75% of original) as the suggested performance benchmark.

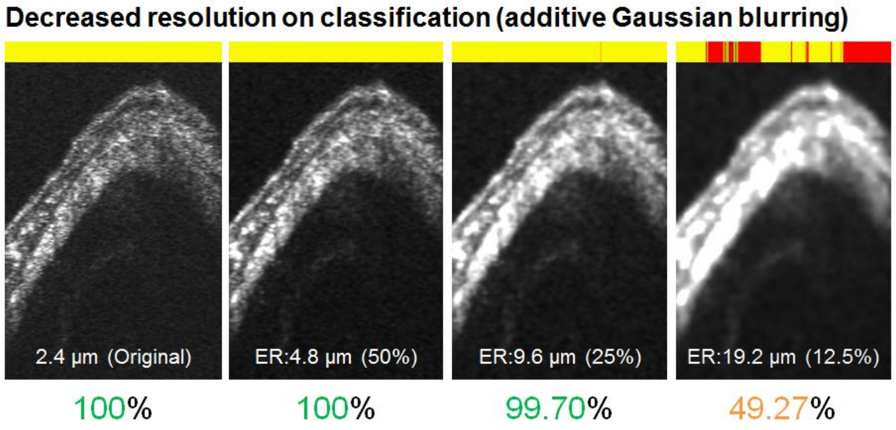

Supplementary Figure 3: Synthetically decreased resolution and classification performance. Using increasing additive Gaussian blurring (from left to right), test images were classified to empirically determine resolution requirements for this platform. Resolution in comparison to the original is inset in each panel. Classification accuracy (shown below each panel) degrades quickly when resolution reaches 12.5% of the original. ER: Effective resolution. Color bar represents predicted class (Green – normal / Yellow – Biofilm / Red – Biofilm and Fluid).

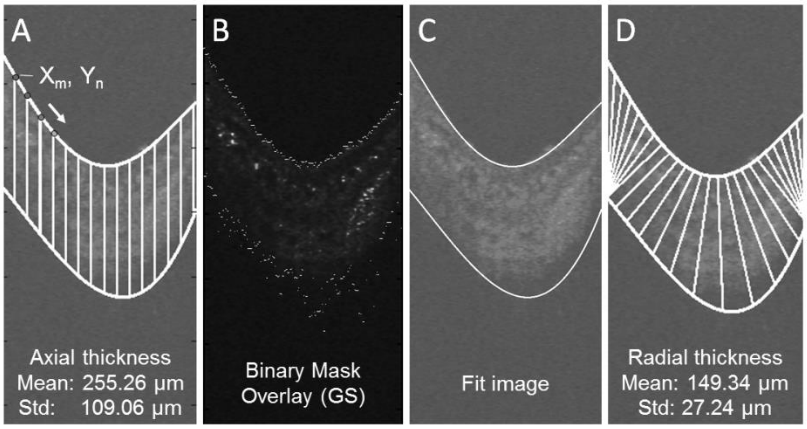

Supplementary Figure 4: Axial and radial thickness. A: Axial fitting and statistics. B: Binary mask with identified points for top and bottom edges. C: Fourth-order polynomial fitting through the points in B. D: Radial thickness fitting and statistics. Radial fitting has a more consistent thickness value across the TM when compared to axial fitting.

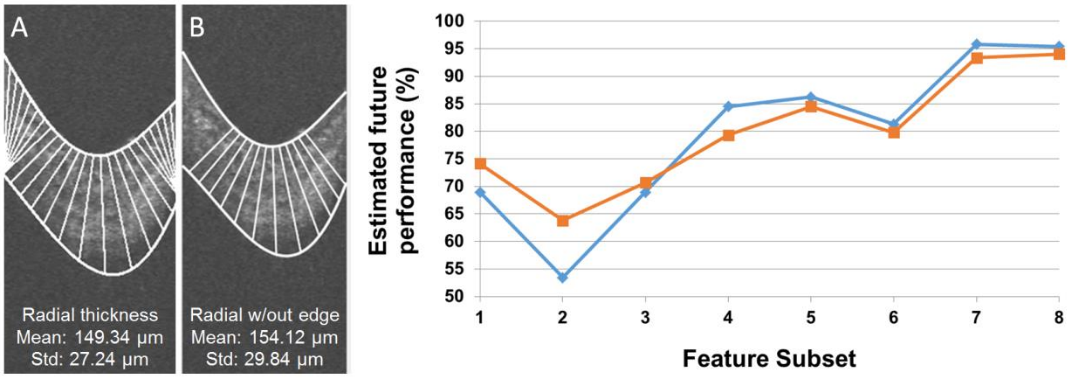

Supplementary Figure 5: Effects of removal of edge A-lines and classifier performance across feature subsets. A: Segmented and fitted image including edge a-lines shows the effect of the program near the edges of the image. B: Segmented and fitted image without the corner points. Fitted lines down sampled for display purposes. Right: Plot comparing the RF-classifier performance with (blue) and without (orange) edge data. Currently, removing the edge cases causes a slight drop in performance, consistent across feature subsets, likely due to the amount of data removed from the classifier database. In the future, as the database grows, edge cases can be safely ignored.

Supplementary Table 1: Clinical findings and consensus labels for OCT. OTO: Fluid identified by the physician using otoscopy, and OCT: fluid identified using OCT data. Discrepancies in fluid identification are bolded. Group # set by OCT reader study. Bilat: Bilateral; ETD: Eustachian tube dysfunction; COME: Chronic otitis media with effusion; RAOM: Recurrent acute otitis media; HL: Hearing loss; URI: Upper respiratory infection; TM: tympanic membrane.

#	Clinical Findings	OTO	OCT	Group
1	Bilat. ETD, mucoid COME	Y	Y	2
2	Bilat. ETD, mucoid COME	Y	Y	2
3	ETD, RAOM	Y	Y	2
4	Bilat. ETD and COME	Y	Y	3
5	No dictation avail.	-	Y	3
6	No dictation avail.	-	Y	3
7	No dictation avail.	-	Y	3
8	No dictation avail.	-	Y	3
9	Bilat. ETD, mucoid COME, HL	Y	Y	2
10	Bilat. ETD, COME	Y	Y	3
11	Bilat. ETD, COME	Y	Y	2
12	Bilat. ETD, COME	Y	Y	2
13	ETD, RAOM	Y	Y	3
14	ETD, RAOM	Y	Y	3
15	RAOM with effusion	Y	Y	3
16	Bilat. COME, RAOM, ETD, HL	Y	Y	3
17	RAOM, hearing loss	Y	Y	2
18	RAOM, hearing loss	Y	Y	2
19	Bilat. ETD, COME	Y	Y	3
20	Bilat. ETD, COME	Y	Y	3
21	Bilat. ETD, COME	Y	Y	3
22	Bilat. ETD, COME	Y	Y	3
23	Bilat. ETD, COME	Y	Y	3
24	ETD, RAOM	Y	Y	3
25	Bilat. ETD, COME	**Y**	**N**	1
26	Bilat. ETD, COME, HL	Y	Y	3
27	Bilat. ETD, COME	**Y**	**N**	1
28	Acute URI (persistent cough)	N	N	1
29	Normal control	N	N	1
30	Normal control	N	N	1
31	Normal control	N	N	1
32	Normal control	N	N	1
33	Normal control	N	N	1
34	Normal control	N	N	1
35	Normal control	N	N	1
36	Normal control	N	N	1
37	Bilat. OME	Y	Y	3
38	Bilat. OME	Y	Y	3
39	Bilat. OME	Y	Y	3
40	Dull TM	N	N	2
41	Normal control	N	N	1
42	Bilat. AOM	Y	Y	3
43	Bilat. AOM	Y	Y	3
44	RAOM	Y	Y	3
45	Normal control	N	N	1
46	Normal control	**N**	**Y**	3
47	No OM complaints	**N**	**Y**	3
48	OME	Y	Y	3
49	Opaque TM	Y	Y	3
50	No OM complaints	**N**	**Y**	3
51	No dictation avail.	-	Y	3
52	Normal control	N	N	1
53	OME	Y	Y	3
54	Dull TM, OME	Y	Y	3
55	AOM	Y	Y	3
56	Normal control	N	N	1
57	Normal control	N	N	1
58	Normal control	N	N	1

Supplementary Table 2: Results from the clinically-focused leave-one B-scan out random forest classification test on Subsets 1-8.

	Feature subsets	Random Forest Leave-one B-scan out CV
1	Clinical report keywords	74.14
2	OMGRADE scale	74.14
3	6 Digital otoscopy metrics (custom)	63.79
4	Physician info (1 + 2)	84.48
5	All Clinical information (1 + 2 + 3)	84.48
6	12 OCT metrics	80.97
7	Clinical & OCT features (5 + 6)	92.19
8	Least useful 5 removed	91.57

Supplementary Table 3: Feature summary table. σ: Standard deviation; atten.: Attenuation; DO: Digital otoscopy; loc: location; MAD: Median absolute distance; Sat: Saturation.

	Feature	Source		Feature	Source
1	Radial thickness	OCT	11	Fourier peak width	OCT
2	σ peak loc	OCT	12	Fourier peak prom.	OCT
3	Mean peak width	OCT	13	OMGRADE	Scale
4	σ peak width	OCT	14	Clinical reports	Keywords
5	Mean peak prom.	OCT	15	Hue, average	DO
6	σ peak prominence	OCT	16	Hue, median	DO
7	Total # peaks	OCT	17	Hue, MAD	DO
8	Optical atten. max	OCT	18	Sat., mean	DO
9	Optical atten mean	OCT	19	Sat., median	DO
10	Optical atten sum	OCT	20	Sat., MAD	DO

Supplementary Table 4: Patient report keywords and point value. OM: Otitis media; TM: Tympanic membrane.

Keyword	Score
Normal ears	0
Unremarkable ears	0
Clear or healthy TM	0
Acute OM	1
Inflammation, erythema, inflammed	1
Fluid or effusion	1
Recent infection	1
Smoke, asthma, allergies	1
Family history of OM	1
Opaque or dull TM	1
Recurrent or chronic OM	2
Antibiotics	2
Referral	2
Persistent or recurrent	2
Purulent, mucoid, mucopurulent	2
